# Navigating the RCOG 2025 and ATA 2026 guideline updates on the management of thyroid disorders during pregnancy

**DOI:** 10.1016/j.crwh.2026.e00827

**Published:** 2026-06-20

**Authors:** Kleoniki I. Athanasiadou, Melpomeni Peppa, Sophia N. Kalantaridou, Stavroula A. Paschou

**Affiliations:** a Endocrine Unit and Diabetes Centre, Department of Clinical Therapeutics, Alexandra Hospital, School of Medicine, National and Kapodistrian University of Athens, Athens, Greece; b Endocrine Unit and Diabetes Centre, Second Department of Internal Medicine, School of Medicine, Attikon University Hospital, National and Kapodistrian University of Athens, Athens, Greece; c 3rd Department of Obstetrics and Gynecology, Attikon University Hospital, School of Medicine, National and Kapodistrian University of Athens, Athens, Greece; d Imperial Centre for Endocrinology, St Mary’s Hospital, Imperial College London, London, UK

**Keywords:** Pregnancy, SCH, Subclinical hypothyroidism, Thyroid, TPOAb

Thyroid disorders are a common endocrine complication of pregnancy. The fetal thyroid gland starts to synthesize thyroxine at 10–12 weeks of gestation, thyroxine secretion starts between 17 and 19 weeks and pituitary control by the fetal thyroid stimulating hormone (TSH) starts at 20 weeks (1). Maternal thyroxine levels are hence vital for the developing fetus, especially during the first trimester of pregnancy. Previous guidelines were more aggressive in suggesting intervention in cases of mild thyroid dysfunction during pregnancy and thyroid autoimmunity.

The latest guideline from the American Thyroid Association (ATA), published in May 2026, constitutes an update of the 2017 guideline (2) and is essentially aligned with that from the Royal College of Obstetricians and Gynaecologists (RCOG) published in July 2025 (3). They both present a new approach in the management of thyroid disorders during pregnancy, especially in hypothyroidism. They move away from the trimester-specific TSH targets of 2.5 mU/L in the first and 3.0 mU/L in second and third trimesters. The use of laboratory-specific thresholds is advised, in the absence of which the diagnostic TSH threshold for hypothyroidism is 4.0 mU/L. Maternal TSH is considered the best marker of thyroid function during pregnancy. Free T4 (fT4) should also be measured.

Diagnostic thresholds and treatment targets are explicitly distinguished in the guidelines. A reasonable treatment target for women with pre-existing hypothyroidism already on levothyroxine replacement should be 0.5–2.5 mU/L. It is advised to use laboratory and trimester-specific TSH and fT4 reference ranges, where available. The use of the same assay during the course of pregnancy will allow for safe comparisons between values and management of analytic variability (4). Women with pre-existing hypothyroidism are advised to self-initiate levothyroxine dose up-titration by 20–30% once they have a positive pregnancy test or simply by doubling the levothyroxine dose for two days each week. By week 20, a dose increase of 50% may be required to remain euthyroid. The dose should return to the pre-pregnancy dose after delivery and thyroid function tests (TFTs) can be reassessed at 6 weeks postpartum.

The guidelines advise against universal thyroid function screening and suggest that only women with risk factors should be offered TSH testing upon pregnancy confirmation (5). Risk factors include history of thyroid disease (subclinical hypothyroidism (SCH) or hyperthyroidism, goiter, thyroid surgery, radioiodine ablation or head and neck ionizing radiation) or thyroid antibody positivity, history of autoimmune diseases (e.g. type 1 diabetes, Addison's disease, pernicious anaemia, vitiligo), Down or Turner syndrome, two or more miscarriages, infertility, use of thyroid-disruptive medications (e.g. amiodarone, lithium, phenytoin, carbamazepine, cancer-related immunotherapy), residence in iodine-deficient areas, and family history of autoimmune thyroid disease [6], [7]. Maternal age, body mass index, and parity have been removed as factors indicating thyroid function testing.

Thyroid antibody positivity seems to be the strongest risk factor and warrants TFTs upon pregnancy confirmation. However, the latest ATA and RCOG guidelines highlight that thyroxine supplementation is not recommended in euthyroid women with positive thyroid peroxidase antibodies (TPOAb) and infertility, recurrent miscarriages or those planning fertility treatment. The double-blind randomized controlled TABLET trial concluded that levothyroxine replacement in euthyroid women with positive TPOAb did not result in higher rate of live births compared with placebo (8). As there is a 7–9% risk of developing overt or subclinical hypothyroidism before or after conception in these women, TFTs should be monitored every 3–6 months preconception. For SCH, repeat testing within 3 weeks is required before treatment start as a high proportion of women will have normal TFTs on remeasurement. The aim of this change is to reduce overtreatment, costs, and patient anxiety. In cases of profound SCH during pregnancy with TSH above 10 mU/L, treatment with levothyroxine is indicated. For mild SCH diagnosed in the first trimester, levothyroxine treatment can be considered after a confirmatory testing within 3 weeks, while for cases diagnosed after the first trimester TSH monitoring every 4–6 weeks is advised. If levothyroxine replacement was started in pregnancy for overt or subclinical hypothyroidism a cessation trial is advised. Follow-up intervals and decision to restart levothyroxine is based on hypothyroid symptoms, TPOAb positivity, and imminent pregnancy plans. It is worth mentioning that in current clinical practice clinicians are commonly prescribing levothyroxine even for mild SCH to maximize clinical benefits; however, this may lead to overmedicalization and patient anxiety. The published trials, like TABLET, and the latest guidelines can be used as evidence-based tools to support clinicians towards less interventional management targeted to women who meet the therapeutic criteria.

For hyperthyroidism the recommendations are largely unchanged. Propylthiouracil (PTU) remains the first-line agent for the first trimester of pregnancy and preconception. Doses should be kept at the lowest effective level. TFTs should be monitored every 2–4 weeks during the first half of pregnancy and every 4–8 weeks after week 20. A new recommendation in the ATA guideline is that urgent thyroid surgery can happen at any time during pregnancy but ideally in the second trimester. Women who underwent surgery can continue breastfeeding as soon as they are awake and able to hold the baby. It is no longer advised to dispose of breast milk for 24 h after anesthesia. In the same context, the guideline recommendations regarding postpartum thyroiditis (PPT) focus on patient education to recognize the hyperthyroid symptoms, aiming to proceed promptly with thyroid testing and treatment with beta blockers for the initial thyrotoxic phase and levothyroxine for the subsequent hypothyroid phase.

Conclusively, the latest guidelines launch substantial changes in the field of thyroid dysfunction during pregnancy aiming to reduce unnecessary thyroid testing and overtreatment, focusing on a patient-centered approach.

## Contributors

Kleoniki I. Athanasiadou contributed drafting the manuscript and undertaking the literature review.

Melpomeni Peppa and Sophia N. Kalantaridou contributed to revising the article critically for important intellectual content.

Stavroula A. Paschou contributed drafting the manuscript, undertaking the literature review and revising the article critically for important intellectual content.

All authors approved the final submitted manuscript.

## Provenance and peer review

This editorial was commissioned and not externally peer reviewed. Stavroula Paschou, an editorial board member of *Case Reports in Women's Health*, was not involved in editorial consideration of the manuscript and was blinded to the process.

## Funding

The authors received no funding from an external source for the publication of this editorial.

## Declaration of competing interest

The authors declare that they have no competing interest regarding the publication of this editorial.
